# Supplementary material for: Machine learning for the detection and diagnosis of cognitive impairment in Parkinson’s Disease: A systematic review
Source: PLoS One. 2024 May 16;19(5):e0303644. doi: 10.1371/journal.pone.0303644 (PMC11098383; doi:10.1371/journal.pone.0303644)
Supplement: S1 File — Search strings used for retrieval of publications from databases. (PDF) [file pone.0303644.s001.pdf]

Machine Learning for the Detection and Diagnosis of Cognitive Impairment in Parkinson's Disease: A Systematic Review - Supplementary Material

Callum Altham*[1], Huaizhong Zhang[1], and Ella Pereira[1]

[1]Department of Computer Science, Edge Hill University, St. Helens Road, Ormskirk, L39 4QP, Lancashire, United Kingdom

Table S1: Search strings used for retrieval of publications from databases.

Database	Search String
PubMed	(("parkinson disease"[MeSH Major Topic]) AND ("cognitive impairment"[MeSH Major Topic] OR cognitive impair*) AND (Machine Learning[MeSH Major Topic] OR machine learn* OR machine-learn* OR deep learn* OR deep-learn*) AND (Diagnosis OR diagnos* OR detect* OR classif* OR identif*[MeSH Major Topic]) NOT (review[Publication Type]))
IEEE Xplore	(Parkinson*) AND (cognitive impairment OR cognitive) AND (machine learning OR machine-learn* OR deep learning OR deep learn* OR deep-learn*) AND (diagnos* OR detect* OR classif* OR identif*)
Scopus	(TITLE-ABS-KEY (parkinson) AND TITLE-ABS-KEY (cognitive AND impairment) OR TITLE-ABS-KEY (cognitive) AND TITLE-ABS-KEY (machine AND learning) OR TITLE-ABS-KEY (machine AND learn*) OR TITLE-ABS-KEY (machine-learn*) OR TITLE-ABS-KEY (deep AND learning) OR TITLE-ABS-KEY (deep AND learn*) OR TITLE-ABS-KEY (deep-learn*) AND TITLE-ABS-KEY (diagnos*) OR TITLE-ABS-KEY (detect*) OR TITLE-ABS-KEY (classif*) OR TITLE-ABS-KEY (identif*)) AND NOT DOCTYPE (re)
Science Direct	("Parkinson") AND ("cognitive impairment") AND ("machine learning" OR "deep learning") AND ("diagnos" OR "detect" OR "classif" OR "identif")

*Corresponding Author: althamc@edgehill.ac.uk
