# Supplementary material for: Machine learning for the detection and diagnosis of cognitive impairment in Parkinson’s Disease: A systematic review
Source: PLoS One. 2024 May 16;19(5):e0303644. doi: 10.1371/journal.pone.0303644 (PMC11098383; doi:10.1371/journal.pone.0303644)
Supplement: S2 File — Secondary search keywords used for retrieval of publications from databases. (PDF) [file pone.0303644.s002.pdf]

Machine Learning for the Detection and Diagnosis of Cognitive Impairment in Parkinson's Disease: A Systematic Review - Supplementary Material

Callum Altham*[1], Huaizhong Zhang[1], and Ella Pereira[1]

[1]Department of Computer Science, Edge Hill University, St. Helens Road, Ormskirk, L39 4QP, Lancashire, United Kingdom

Table S2: Secondary search keywords used for retrieval of publications from databases.

Main Keyword	Secondary Keywords
Parkinson	Parkinson Disease, Parkinson's disease, parkinsonism, idiopathic parkinsonism, primary parkinsonism, Parkinson's disease dementia, Parkinson dementia complex, paralysis agitans
Cognitive Impairment	Cognitive impairments, cognitive dysfunction, cognitive dysfunctions, dysfunction, mild cognitive impairment, MCI, Parkinson's Disease Dementia, PDD, mild cognitive impairments, neurocognitive disorder, cognitive decline, mild neurocognitive disorder, mild cognitive decline, mental deterioration, cognitive disorder, cognitive disorders, cognitive deficit
Machine Learning	Machine learn, machine-learning, machine-learn, deep learning, deep-learn, deep-learning, deep learn, transfer learning, artificial intelligence, AI, risk-prediction, prediction model, decision tree, artificial neural network, ANN, random forest, support vector machine, gradient boosting, linear regression, regression, ensemble learning, k-nearest neighbors

*Corresponding Author: althamc@edgehill.ac.uk
