# Supplementary material for: Machine learning for the detection and diagnosis of cognitive impairment in Parkinson’s Disease: A systematic review
Source: PLoS One. 2024 May 16;19(5):e0303644. doi: 10.1371/journal.pone.0303644 (PMC11098383; doi:10.1371/journal.pone.0303644)
Supplement: S3 File — Referenced Studies, Publication Year, Data Sources, Research Activities, Modalities Employed, Subject Details, Machine Learning Methods, Validation Techniques, and Reported Outcomes. (PDF) [file pone.0303644.s003.pdf]

Machine Learning for the Detection and Diagnosis of Cognitive Impairment in Parkinson's Disease: A Systematic Review - Supplementary Material

Callum Altham*[1], Huaizhong Zhang[1], and Ella Pereira[1]

[1]Department of Computer Science, Edge Hill University, St. Helens Road, Ormskirk, L39 4QP, Lancashire, United Kingdom

Table S3: Summary of Characteristics of Reviewed Literature: Referenced Studies, Publication Year, Data Sources, Research Activities, Modalities Employed, Subject Details, Machine Learning Methods, Validation Techniques, and Reported Outcomes

Reference	Year	Data Source	Activity	Modality	Subjects	Method(s)	Validation	Outcomes
[1]	2017	Collected	Diagnosis	Imaging	Training: 108 (43 PD-NC, 27 PD-MCI, 38 HC); Validation: 25 (17 PD-NC, 8 PD-MCI)	SVM	LOOCV	AUC: 0.81
[2]	2023	Database	Prediction	Demographics, Clinical Characteristics, Imaging, Genetic & Epigenetic, CSF	213	RReliefF + SVR	10-Fold CV	PCC = 0.44
[3]	2022	Collected	Biomarker Identification	Imaging, Gait and Movement	75: (42 No PD-MCI, 33 PD-MCI)	SVM, K-NN, J48 DT, adaBoost, RF	10-Fold CV LOOCV	PD-NC v PD-MCI: PD-MCI patients showed worse gait patterns compared to PD-NC, but no difference was discovered in Amyloid PET; SVM: Accuracy = 80.0% Sensitivity = 72.7% Specificity = 85.7% AUC = 0.792

Continued on the next page

*Corresponding Author: althamc@edgehill.ac.uk

Reference	Year	Data Source	Activity	Modality	Subjects	Method(s)	Validation	Outcomes
[4]	2020	Collected	Diagnosis	Imaging	68<br>(26 PD-NC, 27 PD-MCI, 15 HC)	LoR, SVM, K-NN	50-Fold CV	K-NN:<br>Accuracy = 92.60%<br>Sensitivity = 93%<br>Specificity = 93%
[5]	2021	Collected	Diagnosis	Imaging	51<br>(17 PD-NC, 18 PD-MCI, 16 PDD)	Ensemble DT	LOOCV	PDD vs PD-NC<br>Fine DT:<br>Accuracy = 93.9%<br>Sensitivity = 93.7%<br>Specificity = 94.1%<br><br>PDD vs PD-MCI + PD-NC<br>RUSBoosted Trees:<br>Accuracy = 86.3%<br>Sensitivity = 80%<br>Specificity = 88.9%
[6]	2018	Collected	Diagnosis	EEG	118<br>(28 Cognitively Intact,<br>33 Mental Slowing,<br>43 Mild Cognitive Deficits,<br>14 Severe Deficits)	SVM, K-NN	5-Fold CV	K-NN:<br>Accuracy = 87% +- 2.8
[7]	2022	Collected	Diagnosis	Imaging	116<br>(17 PD-NC,<br>30 Stable MCI,<br>32 PDD Converters,<br>19 PDD,<br>18 HC)	SVM	10-Fold CV	AUC = 0.73<br>Sensitivity = 67%<br>Specificity = 80%
[8]	2022	Collected	Differential Diagnosis	Demographics, Neuropsychological Profile	138<br>(78 Probable PDD,<br>62 Probable DLB)	Binomial LoR,<br>K-NN,<br>SVM, NB,<br>Ensemble	N/D	K-NN:<br>AUC = 0.958<br>Accuracy = 91.2%<br>Sensitivity = 96.42%<br>Specificity = 81%

Continued on the next page

Reference	Year	Data Source	Activity	Modality	Subjects	Method(s)	Validation	Outcomes
[9]	2023	Pre-existing Cohort	Diagnosis	Other	227 (45 PD-NC, 45 PD-MCI, 20 PDD, 11 Other, 106 HC)	Ensemble (SVM, LoR, RF)	Nested 10-Fold CV	AUC = 0.88 Sensitivity = 83% Specificity = 78%
[10]	2020	Database	Diagnosis	Motor Symptoms, Non-Motor Symptoms, Sleep Behaviour	342 (66 EOPDD, 276 PD-NC)	RF, NB, DA	Grid-Search Out-of-bag	RF: Accuracy = 89.5%
[11]	2020	Database	Differential Diagnosis	Sleep Behaviour, Neuropsychological Profile	228 (118 AD, 110 PDD)	RF, LoR, CART	Train-Test Split (70:30)	RF: Accuracy = 73.3% Sensitivity = 78.0% Specificity = 70.0%
[12]	2020	Pre-existing Cohort	Diagnosis	Medical History, Neuropsychological Profile, Other	96 (51 PD-NC, 45 PD-MCI)	RF, DT	Out-of-bag	RF: Accuracy = 65.6% Sensitivity = 70.6% Specificity = 60.0%
[13]	2021	Database	Diagnosis	Clinical Characteristics, Motor Symptoms, Non-Motor Symptoms, Sleep Behaviour	185 (75 PD-NC, 110 PD-MCI)	Hybrid (Polydot, Vanilladot, RBFdot, C5.0)	10-Fold CV	RBFdot + C5.0: AUC = 0.88
[14]	2021	Collected	Diagnosis	Other	368 (48 PD-MCI, 320 HC)	(Undersampling, Oversampling, SMOTE) x (Boosting, Bagging, RF)	5-Fold CV	RF+SMOTE: Accuracy = 74.0%, Sensitivity = 69.2%, Specificity = 75.7%
[15]	2022	Collected	Diagnosis	Imaging	76 (26 PD-NC, 34 PD-MCI, 16 HC)	Fine Gaussian SVM	5-Fold CV	Accuracy = 77.3% Sensitivity = 63.6% Specificity = 69.7%
[16]	2019	Collected	Diagnosis	EEG	70 (43 PD-NC, 27 PD-MCI)	RF	Train-Test Split (70:30) 5-Fold CV (20 Repeats)	AUC = 0.71

Continued on the next page

Reference	Year	Data Source	Activity	Modality	Subjects	Method(s)	Validation	Outcomes
[17]	2020	Collected	Diagnosis	Gait and Movement	81 (47 PD-NC, 34 PD-MCI)	SVM, PCA-SVM	N/D	PCA-SVM: Accuracy = 91.67% AUC = 0.9714
[18]	2022	Collected	Diagnosis	Clinical Characteristics, Blood Biomarkers, Neuropsychological Profile	42 (16 PD-NC, 26 PD-CI)	SVM, PCA-SCM	Train-Test Split (70:30)	PCA-SVM: Accuracy = 92.3%, AUC = 0.929
[19]	2023	Collected	Diagnosis	Imaging	133 (52 PD-NC, 68 PD-MCI)	DT, RF, XGB	Train-Test Split (80:20) 10-Fold CV	XGB: Accuracy = 91.67%, Sensitivity = 92.86%, Specificity = 90%, AUC = 0.94
[20]	2020	Collected	Prediction	Imaging	62 (49 PD-NC, 13 PDD)	CNN	N/D	AUC = 0.81
[21]	2023	Collected	Biomarker Identification	EEG	33 (20 PD-NC, 13 PD-MCI)	NMF	N/D	MCI induces a regularly, interrupted, slow evolution of subnetworks in brain network dynamics of early PD
[22]	2021	Collected	Diagnosis	Blood Biomarkers, Demographics	162 (116 PD, 46 HC)	ANN	Train-Test Split (75:25) 4-Fold CV	Accuracy = 91.3%
[23]	2023	Pre-existing Cohort	Biomarker Identification	Clinical Characteristics, Blood Biomarkers, Genetic & Epigenetic	206 (98 PD-NC, 108 PD-MCI)	ShapleyVIC, Backward Selection, Multivariable log-binomial Regression	N/D	Fewer education years, History of hyper-tension, Higher MDS-UPDRS motor score, Higher TG and ApoA1, Fewer alleles of SNCA associated with increased risk of PD-MCI
[24]	2023	Collected	Diagnosis	Imaging	153 (52 PD-NC, 46 PD-MCI, 20 PDD, 35 HC)	ANN, SVM, RF, GB, K-NN, Ensemble	5-Fold Stratified CV (10 Repeats)	SVM + FD features Accuracy 78 ± 1% Precision = 0.80 Recall = 0.80
[25]	2021	Collected	Biomarker Identification	Speech Features	80 (24 PD-NC, 16 PD-MCI, 40 HC)	SVM	Nested 5-Fold CV	Highest with Phonemic Identifiability during retelling Accuracy 72.1%

Continued on the next page

Reference	Year	Data Source	Activity	Modality	Subjects	Method(s)	Validation	Outcomes
[26]	2022	Collected	Diagnosis	Speech Feature	80<br>(24 PD-NC,<br>16 PD-MCI,<br>40 HC)	SVM	Nested 5-Fold CV	Action Text:<br>Accuracy = 63.3%<br>Sensitivity = 20.0%<br>Specificity = 90.0%<br>F-Score = 48.0%<br><br>Non Action Text<br>Accuracy = 60.5%<br>Sensitivity = 40.0%<br>Specificity = 71.7%<br>F-Score = 50.3%
[27]	2021	Collected	Diagnosis	EEG	40<br>(20 High Cognitive Score<br>20 Low Cognitive Score)	RF + Bayesian Optimisation	10-Fold CV<br>Split Sample Validation	Accuracy = 92%<br>Sensitivity = 90%<br>Specificity = 94%
[28]	2021	Collected	Diagnosis	EEG	43<br>(25 PD-NC, 17 PD-MCI)	RF + Bayesian Optimisation	10-Fold CV	Accuracy 88%<br>Sensitivity = 95%<br>Specificity = 76%<br>F Score = 84%
[29]	2022	Database	Diagnosis	Clinical Characteristics<br>Genetic & Epigenetic<br>Other	209<br>(67 PD-NC<br>60 PD-Subjective Cognitive Decline<br>39 PD-MCI<br>43 PDD)	RF<br>ElasticNet<br>SVM<br>CIF	10-Fold CV<br>(10 Repeats)	CIF:<br>Accuracy = 84.0%<br>Sensitivity = 71.90%<br>Specificity = 96.10%<br>MCC = 0.721<br>AUC = 0.938
[30]	2018	Database	Diagnosis	Medical History, Sleep Behaviours	351<br>(313 PD-NC, 38 PD-MCI)	LoR	Bootstrap Resampling<br>(1000 Iterations)	Accuracy = 74.90%

Continued on the next page

Reference	Year	Data Source	Activity	Modality	Subjects	Method(s)	Validation	Outcomes
[31]	2023	Database	Diagnosis	Imaging, Clinical Characteristics	297	AdaBoost<br>Bagging<br>Gradient Boosting<br>RF<br>XGB<br>MLP<br>K-NN<br>ETC<br>CNN<br>Combined with ANOVA	Train-Test Split (80:20)<br>5-Fold CV	ANOVA + ETC:<br>Accuracy = 78% ± 9%
[32]	2024	Database	Prediction	Imaging<br>Clinical Characteristics	90<br>(57 PD-MCI Converters<br>33 PD-MCI non-converters)	LDA, SVM, K-NN, NB	Bootstrap Re-sampling<br>(100 Iterations)	LDA:<br>AUC = 0.85<br>Accuracy = 85%<br>Sensitivity = 86%<br>Specificity = 84%
[33]	2021	Database	Diagnosis	Neuropsychological Profile	467	DT, SVM, K-NN, NB, RF, MLP	10-Fold CV	MLP:<br>Accuracy = 97.50%<br>Precision = 97.50%<br>Recall = 97.50%<br>F-Score = 97.50%<br>AUC = 0.995
[34]	2022	Collected	Differential Diagnosis	EEG	80<br>(32 AD, 26 DLB, 22 PDD)	K-NN	10-Fold CV	Accuracy = 61 ± 16%<br>Sensitivity = 30 ± 22%<br>Specificity = 76 ± 20%<br>AUC = 0.61
[35]	2022	Database	Diagnosis	Neuropsychological Profile	397	LoR, SVM, RF	Train-Test Split (80:20)	RF:<br>Accuracy = 88%<br>Sensitivity = 89%<br>Specificity = 87%<br>PPV = 0.88<br>NPV = 0.88

Continued on the next page

Reference	Year	Data Source	Activity	Modality	Subjects	Method(s)	Validation	Outcomes
[36]	2022	Collected	Diagnosis	Imaging	149 (22 PD-NC, 26 PD-MCI, 56 PDD, 45 HC)	Multivariable LoR, SVM	Train-Test Split (70:30)	SVM: AUC = 0.95
[37]	2022	Database	Diagnosis	Clinical Characteristics, Imaging, CSF	165	SVM, RF	Train-Test Split Out-of-bag	SVM: MAE = 0.076 RMSE = 0.542
[38]	2019	Collected	Diagnosis	EEG	40 (20 PD-NC, 20 PD-CI)	RF	10-Fold CV	Accuracy = 91.0% ± 2.2 F Score = 92.1% ± 2.1 Precision = 90.25% ± 2.8 Recall = 94.0% ± 2.2 AUC = 0.98 ± 0.011
[39]	2022	Collected	Prediction	Imaging	55 (19 PD-NC, 46 PD-MCI)	RF	Train-Test Split (80:30) (1000 Repeats)	RMSE 11.28 ± 9.51%
[40]	2021	Collected	Diagnosis	Imaging	179 (72 PD-NC, 59 PD-MCI, 48 HC)	RF	10-Fold CV (100 Repeats)	Accuracy = 85.2 ± 7.4% Cohen's = 0.63 ± 0.18 Sensitivity = 88.1 ± 7.9% Specificity = 82.6 ± 7.0%
[41]	2017	Collected	Prediction	Clinical Characteristics Genetic & Epigenetic	Training: 1350 Testing: 1132	Cox Regression	N/D	CI: AUC = 0.85 PDD: AUC = 0.88 NPV = 0.92
[42]	2019	Pre-existing Cohort	Prediction	Clinical Characteristics, Other	237	RF	10-Fold CV (10 Repeats) LOOCV	AUC = 0.97

Continued on the next page

Reference	Year	Data Source	Activity	Modality	Subjects	Method(s)	Validation	Outcomes
[43]	2023	Collected	Prediction	Demographics<br>Gait & Movement<br>Blood Biomarkers<br>Genetic & Epigenetic<br>Clinical Characteristics<br>Neuropsychological Profile	48<br>(34 PD-NC, 14 PD-ID)	RF	3-Fold Stratified CV	AUC = 0.85<br>Accuracy = 81%<br>Precision = 81%<br>Recall = 51%<br>nMCC = 0.76<br>F Score = 60%
[44]	2013	Collected	Diagnosis	Imaging	45<br>(16 PD-NC,<br>15 PD-MCI,<br>14 PDD)	NB, SVM	5-Fold Stratified CV	Filter Selective NB:<br>Accuracy = 70 ± 26.66<br>Sensitivity = 70 ± 26.66<br>Specificity = 85.56 ± 8.42
[45]	2022	Collected	Diagnosis	Neuropsychological Profile	500	NB, QDA, LoR, Ridge, LDA, ETC, RF, AdaBoost, CatBoost, DT, LGB, XGB, K-NN, SVM	Train-Test Split<br>(70:30)<br>10-Fold CV	QDA:<br>Accuracy = 68.20%<br>AUC = 0.81<br>Sensitivity/Recall = 68.20%<br>Precision = 68.10%<br>F-Score = 67.60%<br>Cohen's = 0.508<br>MCC = 0.513
[46]	2023	Database	Diagnosis	Imaging, Clinical Characteristics	134<br>(47 Stable, 87 Declining)	DNN	Train-Validation-Test Split<br>(60:20:20)<br>3-Fold CV	AUC = 0.81
[47]	2023	Collected	Diagnosis	EEG	36<br>(20 PD-NC, 16 PD-MCI)	DNN	4-Fold CV	Accuracy = 99.41%<br>Sensitivity = 98.85%<br>Specificity = 99.0%<br>AUC = 0.99<br>QWK = 0.98<br>**Average Calculated by Author**
[48]	2023	Collected	Diagnosis	EEG	36<br>(20 PD-NC, 16 PD-MCI)	DNN	Train-Test Split<br>(50:50)<br>4-Fold CV	Accuracy = 99.72%<br>Sensitivity = 99.40%<br>Specificity = 99.58%<br>AUC = 0.99<br>QWK = 0.98<br>**Average Calculated by Author**

Continued on the next page

Reference	Year	Data Source	Activity	Modality	Subjects	Method(s)	Validation	Outcomes
[49]	2023	Collected	Prediction	Imaging, Clinical Characteristics	262<br>(187 No PDD, 75 PDD)	ETC	10-Fold CV	AUC = 0.89<br>Accuracy = 79.80%<br>Sensitivity = 75.0%<br>Specificity = 81.40%
[50]	2016	Database	Diagnosis	Imaging	109<br>(55 PD-NC,<br>22 PD-MCI, 32 HC)	SVM	10-Fold CV	Accuracy = 80.84%<br>Sensitivity = 77.05%<br>Specificity = 84.57%<br>AUC = 0.8677
[51]	2017	Collected	Diagnosis	Gait & Movement	85<br>(22 PD-NC, 23 PD-MCI, 10 PDD, 30 HC)	CGP, SVM, ANN	5-Fold CV	PD-NC vs PD-MCI:<br>SVM<br>AUC = 0.78<br><br>PD-NC vs PDD<br>CGP<br>AUC = 0.83
[52]	2021	Collected	Differential Diagnosis	Imaging	117<br>(30 MCI, 27 PDD,<br>27 AD, 33 HC)	DT, RF, NB, MLP, SVM	Train-Test Split (66:33)<br>5-Fold CV	SVM:<br>Precision = 77.60%<br>Recall = 77.60%<br>F Score = 77.60%<br>AUC = 0.892
[53]	2021	Collected	Prediction	Imaging,<br>Genetic &<br>Epigenetic,<br>Clinical<br>Characteristics,<br>Demographics	101	RReliefF + SVR	Nested LOOCV	11 features were found to be predictive of global cognition in PD.<br><br>A robust association between the rs894280 of SNCA gene and global cognition.<br><br>Correlation Coefficient, r2 = 0.54, 0.29
[54]	2020	Collected	Diagnosis	Gait & Movement	45<br>(22 PD-NC, 23 PD-MCI)	DT, RF, K-NN	LOOCV	DT:<br>Accuracy = 86.8%,<br>Sensitivity = 85.3%<br>Specificity = 88.2%<br>ROC = 0.841
[55]	2023	Collected	Diagnosis	Gait & Movement	80<br>(40 PD-NC, 40 PD-MCI)	DT, RF, NB, SVM, K-NN	External Validation Dataset<br>LOOCV	RF:<br>Accuracy = 81%<br>Sensitivity = 60%<br>Specificity = 100%

Continued on the next page

Reference	Year	Data Source	Activity	Modality	Subjects	Method(s)	Validation	Outcomes
[56]	2019	Database	Prediction	Motor Symptoms, Demographics, Clinical Characteristics, Neuropsychological Profile, Medical History, Other	492	LLMT, RBF, MLP, LASSO-LAR, RF, RNN, BRR, DT, PAR, TSR	Train-Validation-Test Split (65:5:30) (10 Repeats)	LLMT + GP: MAE = 1.68 ± 0.12
[57]	2017	Database	Prediction	Clinical Characteristics, Genetic & Epigenetic, CSF, Imaging	568 (390 PD, 178 HC)	LoR	10-Fold CV Cohort Splitting	AUC = 0.80 R2 = 0.20
[58]	2022	Collected	Diagnosis	Imaging	163 (80 PD-NC, 83 PD-MCI)	RF, XGB, LGB	10-Fold CV Grid Search with Nested 10-Fold CV	RF: Accuracy = 79.1% Sensitivity = 77.3% Specificity = 81.0% AUC = 0.78
[59]	2021	Collected	Prediction	Imaging	141 (46 PDD Converters, 95 PDD Converters)	RF, SVM	10-Fold CV External Validation Dataset	RF: AUC = 0.84
[60]	2021	Collected	Diagnosis	Imaging, Neuropsychological Profile	70 (17 PD-NC, 24 PD-MCI, 29 HC)	SVM	5-Fold Stratified CV	PD-MCI vs HC: Accuracy = 85.33% Sensitivity = 84.00% Specificity = 86.67% PD-NC vs PD-MCI: Accuracy = 64.67% Sensitivity = 53.33% Specificity = 76.00%
[61]	2023	Collected	Biomarker Identification	Imaging	27 (10 PD-NC, 4 PD-MCI, 4 PDD, 9 HC)	CNN	Patient Level LOOCV	Severity of cognitive impairment can be assessed based on brain areas identified by CNN activation maps
[62]	2022	Collected	Diagnosis	Gait & Movement, Speech Features	17 (9 PD-NC, 8 PD-MCI)	GMM	LOOCV	AUC = 0.84

Continued on the next page

Reference	Year	Data Source	Activity	Modality	Subjects	Method(s)	Validation	Outcomes
[63]	2023	Collected	Diagnosis	Gait & Movement	89<br>(52 PD-NC, 10 PDD, 27 HC)	RF	5-Fold CV	Accuracy = 97.5%<br>AUC = 0.95
[64]	2019	Database	Diagnosis	Medical History<br>Clinical Characteristics<br>Motor Symptoms<br>Non-Motor Symptoms<br>Imaging<br>Genetic & Epigenetic<br>Other	333<br>(270 PD-NC, 63 PD-MCI or PDD)	DT + AdaBoost	5-Fold CV	Accuracy = 80.38%<br>Sensitivity = 78.17%<br>Specificity = 82.46%
[65]	2023	Collected	Diagnosis	EEG	36<br>(15 Mild PDD,<br>2 Moderate PDD, 19 HC)	LDA	10-Fold CV<br>(5 Repeats)	Accuracy = 94%<br>Precision = 96%<br>Recall = 95%
[66]	2023	Collected	Biomarker Identification	Imaging	245<br>(195 PD, 25 PD-NC, 25 PD-MCI)	DNN	5-Fold CV<br>Train-Test Split (80:20)	Left parasubiculum, left HATA, and left pre-subiculum could be important biomarkers for conversion from PD-NC to PD-MCI
[67]	2023	Collected	Diagnosis	Imaging	100<br>(40 IPD-NCI, 30 IPD-MCI, 30 HC)	SVM	Train-Test Split (70:30)<br>10-Fold CV	Accuracy = 79%<br>Sensitivity = 78%<br>Specificity = 75%<br>AUC = 0.80
[68]	2020	Collected	Diagnosis	Imaging	66<br>(22 PD-NC,<br>22 PD-NC, 22 HC)	SVM	LOOCV<br>10-Fold CV	Accuracy = 86.36%<br>Sensitivity = 90.91%<br>Specificity = 81.82%
[69]	2020	Collected	Diagnosis	Imaging	93<br>(35 PD-NC, 58 PD-MCI, 20 HC)	SVM	Train-Test Split (80:20)<br>5-Fold CV<br>LOOCV	Accuracy = 80%<br>Sensitivity = 85%<br>Specificity = 71%<br>PPV = 0.85<br>NPV = 0.71<br>MCC = 0.56
[70]	2021	Collected	Diagnosis	EEG, Imaging	71<br>(35 PD-NC, 36 PD-MCI)	SVM	Train-Test Split (80:20)<br>5-Fold CV	Accuracy = 80%<br>Sensitivity = 78%<br>Specificity = 83%<br>AUC = 0.77

S3 Legend:

- **AD:** Alzheimer's Disease
- **ANN:** Artificial Neural Network
- **ANOVA:** Analysis of Variance
- **AUC:** Area under the ROC Curve
- **BRR:** Bayesian Ridge Regression
- **CART:** Classification and Regression Tree
- **CGP:** Cartesian Genetic Programming
- **CI:** Cognitive Impairment
- **CIF:** Conditional Inference Forest
- **CNN:** Convolutional Neural Network
- **CSF:** Cerebrospinal Fluid
- **CV:** Cross Validation
- **DA:** Discriminant Analysis
- **DLB:** Dementia with Lewy Bodies
- **DNN:** Deep Neural Network
- **DT:** Decision Tree
- **EEG:** Electroencephalogram
- **EOPDD:** Early Onset PDD
- **ETC:** Extra Trees Classifier
- **FD:** Higuchi's Fractal Dimension
- **GMM:** Gaussian Mixture Model
- **GP:** Genetic Programming
- **HC:** Healthy Controls
- **IPD:** Incipient Parkinson's Disease
- **K-NN:** K Nearest Neighbour
- **LASSO-LAR:** Least Absolute Shrinkage and Selection Operator – Least Angle Regression
- **LDA:** Linear Discriminant Analysis
- **LGB:** Light Gradient Boosting
- **LiR:** Linear Regression
- **LLMT:** Local Linear Model Trees
- **LOOCV:** Leave One Out Cross Validation
- **LoR:** Logistic Regression
- **MAE:** Mean Absolute Error
- **MCC:** Matthews Correlation Coefficient
- **MCI:** Mild Cognitive Impairment
- **MLP:** Multi-Layer Perceptron
- **N/D:** Not Discussed
- **NB:** Naïve Bayes
- **nMCC:** Normalised Matthews Correlation Coefficient
- **NMF:** Non-Negative Matrix Factorisation
- **NPV:** Negative Predictive Value
- **PAR:** Passive Aggressive Regression
- **PCA:** Principal Component Analysis
- **PCC:** Pearson's Correlation Coefficient
- **PD:** Parkinson's Disease
- **PD-CI:** Parkinson's Disease with Cognitive Impairment
- **PDD:** Parkinson's Disease Dementia
- **PD-MCI:** Parkinson's Disease with Mild Cognitive Impairment
- **PD-NC:** Parkinson's Disease with Normal Cognition
- **PET:** Positron Emission Tomography
- **PPV:** Positive Predictive Value
- **QDA:** Quadratic Discriminant Analysis
- **QWK:** Quadratic Weighted Kappa
- **RBF:** Radial Basis Function
- **RF:** Random Forest
- **RMSE:** Root Mean Square Error
- **RNN:** Recurrent Neural Network
- **SMOTE:** Synthetic Minority Over-sampling Technique
- **SVM:** Support Vector Machine
- **SVR:** Support Vector Regression
- **TSR:** Thiel-Sen Regression
- **XGB:** XGBoost

References

[1] Abós A, Baggio HC, Segura B, García-Díaz AI, Compta Y, Martí MJ, et al. Discriminating Cognitive Status in Parkinson's Disease through Functional Connectomics and Machine Learning. Scientific Reports. 2017;7(1):1–13. doi:10.1038/srep45347.

[2] Almgren H, Camacho M, Hanganu A, Kibreab M, Camicioli R, Ismail Z, et al. Machine Learning-Based Prediction of Longitudinal Cognitive Decline in Early Parkinson's Disease Using Multimodal Features. Scientific Reports. 2023;13(1):1–9. doi:10.1038/s41598-023-37644-6.

[3] Amboni M, Ricciardi C, Adamo S, Nicolai E, Volzone A, Erro R, et al. Machine Learning Can Predict Mild Cognitive Impairment in Parkinson's Disease. Frontiers in Neurology. 2022;13. doi:10.3389/fneur.2022.1010147.

[4] Arslan DB, Gurvit H, Genc O, Kicik A, Eryurek K, Cengiz S, et al. The Cerebral Blood Flow Deficits in Parkinson's Disease with Mild Cognitive Impairment Using Arterial Spin Labeling MRI. Journal of Neural Transmission. 2020;127(9):1285–1294. doi:10.1007/S00702-020-02227-6.

[5] Azamat S, Arslan DB, Erdogdu E, Kicik A, Cengiz S, Eryürek K, et al. Detection of Visual and Frontoparietal Network Perfusion Deficits in Parkinson's Disease Dementia. European Journal of Radiology. 2021;144. doi:10.1016/j.ejrad.2021.109985.

[6] Betrouni N, Delval A, Chaton L, Defebvre L, Duits A, Moonen A, et al. Electroencephalography-Based Machine Learning for Cognitive Profiling in Parkinson's Disease: Preliminary Results. Movement Disorders. 2019;34(2):210–217. doi:10.1002/MDS.27528.

[7] Booth S, Park KW, Lee CS, Ko JH. Predicting Cognitive Decline in Parkinson's Disease Using FDG-PET–Based Supervised Learning. The Journal of Clinical Investigation. 2022;132(20). doi:10.1172/JCI157074.

[8] Bougea A, Efthymiopoulou E, Spanou I, Zikos P. A Novel Machine Learning Algorithm Predicts Dementia With Lewy Bodies Versus Parkinson's Disease Dementia Based on Clinical and Neuropsychological Scores. Journal of Geriatric Psychiatry and Neurology. 2022;35(3):317–320. doi:10.1177/0891988721993556.

[9] Brien DC, Riek HC, Yep R, Huang J, Coe B, Areshenkoff C, et al. Classification and Staging of Parkinson's Disease Using Video-Based Eye Tracking. Parkinsonism and Related Disorders. 2023;110. doi:10.1016/j.parkreldis.2023.105316.

[10] Byeon H. Best Early-Onset Parkinson Dementia Predictor Using Ensemble Learning among Parkinson's Symptoms, Rapid Eye Movement Sleep Disorder, and Neuropsychological Profile. World journal of psychiatry. 2020;10(11):245–259. doi:10.5498/wjp.v10.i11.245.

[11] Byeon H. Application of Machine Learning Technique to Distinguish Parkinson's Disease Dementia and Alzheimer's Dementia: Predictive Power of Parkinson's Disease-Related Non-Motor Symptoms and Neuropsychological Profile. Journal of Personalized Medicine. 2020;10(2):31. doi:10.3390/JPM10020031.

[12] Byeon H. Is the Random Forest Algorithm Suitable for Predicting Parkinson's Disease with Mild Cognitive Impairment out of Parkinson's Disease with Normal Cognition? International Journal of Environmental Research and Public Health 2020, Vol 17, Page 2594. 2020;17(7):2594. doi:10.3390/IJERPH17072594.

[13] Byeon H. Development of a Predictive Model for Mild Cognitive Impairment in Parkinson's Disease with Normal Cognition Using Kernel-Based C5.0 Machine Learning Blending: Preliminary Research. Engineering Proceedings. 2021;11(1):18. doi:10.3390/ASEC2021-11147/S1.

[14] Byeon H. Exploring Parkinson's Disease Predictors Based on Basic Intelligence Quotient and Executive Intelligence Quotient. International Journal of Advanced Computer Science and Applications. 2021;12(4):106–111. doi:10.14569/IJACSA.2021.0120414.

[15] Cengiz S, Arslan DB, Kicik A, Erdogdu E, Yildirim M, Hatay GH, et al. Identification of Metabolic Correlates of Mild Cognitive Impairment in Parkinson's Disease Using Magnetic Resonance Spectroscopic Imaging and Machine Learning. Magnetic Resonance Materials in Physics, Biology and Medicine. 2022;35(6):997–1008. doi:10.1007/S10334-022-01030-6.

[16] Chaturvedi M, Bogaarts JG, Kozak (Cozac) VV, Hatz F, Gschwandtner U, Meyer A, et al. Phase Lag Index and Spectral Power as QEEG Features for Identification of Patients with Mild Cognitive Impairment in Parkinson's Disease. Clinical Neurophysiology. 2019;130(10):1937–1944. doi:10.1016/J.CLINPH.2019.07.017.

[17] Chen PH, Lien CW, Wu WC, Lee LS, Shaw JS. Gait-Based Machine Learning for Classifying Patients with Different Types of Mild Cognitive Impairment. Journal of Medical Systems. 2020;44(6):1–6. doi:10.1007/s10916-020-01578-7.

[18] Chen PH, Hou TY, Cheng FY, Shaw JS, Ferrucci R, Chen PH, et al. Prediction of Cognitive Degeneration in Parkinson's Disease Patients Using a Machine Learning Method. Brain Sciences. 2022;12(8):1048. doi:10.3390/BRAINSCI12081048.

[19] Chen B, Xu M, Yu H, He J, Li Y, Song D, et al. Detection of Mild Cognitive Impairment in Parkinson's Disease Using Gradient Boosting Decision Tree Models Based on Multilevel DTI Indices. Journal of Translational Medicine. 2023;21(1):1–11. doi:10.1186/S12967-023-04158-8.

[20] Choi H, Kim YK, Yoon EJ, Lee JY, Lee DS. Cognitive Signature of Brain FDG PET Based on Deep Learning: Domain Transfer from Alzheimer's Disease to Parkinson's Disease. European Journal of Nuclear Medicine and Molecular Imaging. 2020;47(2):403–412. doi:10.1007/S00259-019-04538-7.

[21] Chu C, Zhang Z, Wang J, Wang L, Shen X, Bai L, et al. Evolution of Brain Network Dynamics in Early Parkinson's Disease with Mild Cognitive Impairment. Cognitive Neurodynamics. 2023;17(3):681–694. doi:10.1007/S11571-022-09868-1.

[22] Chung CC, Chan L, Chen JH, Bamodu OA, Chiu HW, Hong CT. Plasma Extracellular Vesicles Tau and β-Amyloid as Biomarkers of Cognitive Dysfunction of Parkinson's Disease. The FASEB Journal. 2021;35(10):e21895. doi:10.1096/FJ.202100787R.

[23] Deng X, Ning Y, Saffari SE, Xiao B, Niu C, Ng SYE, et al. Identifying Clinical Features and Blood Biomarkers Associated with Mild Cognitive Impairment in Parkinson Disease Using Machine Learning. European Journal of Neurology. 2023;30(6):1658–1666. doi:10.1111/ENE.15785.

[24] Fiorenzato E, Moaveninejad S, Weis L, Biundo R, Antonini A, Porcaro C. Brain Dynamics Complexity as a Signature of Cognitive Decline in Parkinson's Disease. Movement Disorders. 2024;39(2):305–317. doi:10.1002/mds.29678.

[25] García AM, Arias-Vergara T, Vasquez-Correa JC, Nöth E, Schuster M, Welch AE, et al. Cognitive Determinants of Dysarthria in Parkinson's Disease: An Automated Machine Learning Approach. Movement Disorders. 2021;36(12):2862–2873. doi:10.1002/MDS.28751.

[26] García AM, Escobar-Grisales D, Correa JCV, Bocanegra Y, Moreno L, Carmona J, et al. Detecting Parkinson's Disease and Its Cognitive Phenotypes via Automated Semantic Analyses of Action Stories. npj Parkinson's Disease. 2022;8(1):1–10. doi:10.1038/s41531-022-00422-8.

[27] Geraedts VJ, Koch M, Contarino MF, Middelkoop HAM, Wang H, van Hilten JJ, et al. Machine Learning for Automated EEG-based Biomarkers of Cognitive Impairment during Deep Brain Stimulation Screening in Patients with Parkinson's Disease. Clinical Neurophysiology. 2021;132(5):1041–1048. doi:10.1016/J.CLINPH.2021.01.021.

[28] Geraedts VJ, Koch M, Kuiper R, Kefalas M, Bäck THW, van Hilten JJ, et al. Preoperative Electroencephalography-Based Machine Learning Predicts Cognitive Deterioration After Subthalamic Deep Brain Stimulation. Movement Disorders. 2021;36(10):2324–2334. doi:10.1002/MDS.28661.

[29] Harvey J, Reijnders RA, Cavill R, Duits A, Köhler S, Eijssen L, et al. Machine Learning-Based Prediction of Cognitive Outcomes in de Novo Parkinson's Disease. npj Parkinson's Disease. 2022;8(1):1–11. doi:10.1038/s41531-022-00409-5.

[30] Hogue O, Fernandez HH, Floden DP. Predicting Early Cognitive Decline in Newly-Diagnosed Parkinson's Patients: A Practical Model. Parkinsonism and Related Disorders. 2018;56:70–75. doi:10.1016/j.parkreldis.2018.06.031.

[31] Hosseinzadeh M, Gorji A, Jouzdani AF, Rezaeijo SM, Rahmim A, Salmanpour MR. Prediction of Cognitive Decline in Parkinson's Disease Using Clinical and DAT SPECT Imaging Features, and Hybrid Machine Learning Systems. Diagnostics. 2023;13(10):1691. doi:10.3390/DIAGNOSTICS13101691.

[32] Huang X, He Q, Ruan X, Li Y, Kuang Z, Wang M, et al. Structural Connectivity from DTI to Predict Mild Cognitive Impairment in de Novo Parkinson's Disease. NeuroImage: Clinical. 2024;41:103548. doi:10.1016/j.nicl.2023.103548.

[33] Ismail NH, Kamarudin NS, Nasir AFA. The Neuropsychology Assessment for Identifying Dementia in Parkinson's Disease Patients Using a Deep Neural Network. In: 2021 International Conference on Software Engineering and Computer Systems and 4th International Conference on Computational Science and Information Management, ICSECS-ICOCSIM 2021. Institute of Electrical and Electronics Engineers Inc.; 2021. p. 238–243.

[34] Jennings JL, Peraza LR, Baker M, Alter K, Taylor JP, Bauer R. Investigating the Power of Eyes Open Resting State EEG for Assisting in Dementia Diagnosis. Alzheimer's Research and Therapy. 2022;14(1):1–12. doi:10.1186/S13195-022-01046-Z.

[35] Jeon J, Kim K, Baek K, Chung SJ, Yoon J, Kim YJ. Accuracy of Machine Learning Using the Montreal Cognitive Assessment for the Diagnosis of Cognitive Impairment in Parkinson's Disease. Journal of Movement Disorders. 2022;15(2):132–139. doi:10.14802/JMD.22012.

[36] Kang JJ, Chen Y, Xu GD, Bao SL, Wang J, Ge M, et al. Combining Quantitative Susceptibility Mapping to Radiomics in Diagnosing Parkinson's Disease and Assessing Cognitive Impairment. European Radiology. 2022;32(10):6992–7003. doi:10.1007/S00330-022-08790-8.

[37] Kibtia H, Abdullah S, Bustamam A. Comparison of Random Forest and Support Vector Machine for Prediction of Cognitive Impairment in Parkinson's Disease. AIP Conference Proceedings. 2020;2296:20093. doi:10.1063/5.0030332.

[38] Koch M, Geraedts V, Wang H, Tannemaat M, Back T. Automated Machine Learning for EEG-Based Classification of Parkinson's Disease Patients. In: Proceedings - 2019 IEEE International Conference on Big Data, Big Data 2019. Institute of Electrical and Electronics Engineers Inc.; 2019. p. 4845–4852.

[39] Kübler D, Wellmann SK, Kaminski J, Skowronek C, Schneider GH, Neumann WJ, et al. Nucleus Basalis of Meynert Predicts Cognition after Deep Brain Stimulation in Parkinson's Disease. Parkinsonism and Related Disorders. 2022;94:89–95. doi:10.1016/j.parkreldis.2021.12.002.

[40] Lin H, Liu Z, Yan W, Zhang D, Liu J, Xu B, et al. Brain Connectivity Markers in Advanced Parkinson's Disease for Predicting Mild Cognitive Impairment. European Radiology. 2021;31(12):9324–9334. doi:10.1007/S00330-021-08086-3.

[41] Liu G, Locascio JJ, Corvol JC, Boot B, Liao Z, Page K, et al. Prediction of Cognition in Parkinson's Disease with a Clinical–Genetic Score: A Longitudinal Analysis of Nine Cohorts. The Lancet Neurology. 2017;16(8):620–629. doi:10.1016/S1474-4422(17)30122-9.

[42] Lo C, Arora S, Baig F, Lawton MA, Mouden CE, Barber TR, et al. Predicting Motor, Cognitive & Functional Impairment in Parkinson's. Annals of Clinical and Translational Neurology. 2019;6(8):1498–1509. doi:10.1002/ACN3.50853.

[43] McFall GP, Bohn L, Gee M, Drouin SM, Fah H, Han W, et al. Identifying Key Multi-Modal Predictors of Incipient Dementia in Parkinson's Disease: A Machine Learning Analysis and Tree SHAP Interpretation. Frontiers in Aging Neuroscience. 2023;15:1124232. doi:10.3389/FNAGI.2023.1124232.

[44] Morales DA, Vives-Gilabert Y, Gómez-Ansón B, Bengoetxea E, Larrañaga P, Bielza C, et al. Predicting Dementia Development in Parkinson's Disease Using Bayesian Network Classifiers. Psychiatry Research: Neuroimaging. 2013;213(2):92–98. doi:10.1016/J.PSCYCHRESNS.2012.06.001.

[45] Ortelli P, Ferrazzoli D, Versace V, Cian V, Zarucchi M, Gusmeroli A, et al. Optimization of Cognitive Assessment in Parkinsonisms by Applying Artificial Intelligence to a Comprehensive Screening Test. npj Parkinson's Disease. 2022;8(1):1–9. doi:10.1038/s41531-022-00304-z.

[46] Ostertag C, Visani M, Urruty T, Beurton-Aimar M. Long-Term Cognitive Decline Prediction Based on Multi-Modal Data Using Multimodal3DSiameseNet: Transfer Learning from Alzheimer's Disease to Parkinson's Disease. International Journal of Computer Assisted Radiology and Surgery. 2023;18(5):809–818. doi:10.1007/S11548-023-02866-6.

[47] Parajuli M, Amara AW, Shaban M. Deep-Learning Detection of Mild Cognitive Impairment from Sleep Electroencephalography for Patients with Parkinson's Disease. PLOS ONE. 2023;18(8):e0286506. doi:10.1371/JOURNAL.PONE.0286506.

[48] Parajuli M, Amara AW, Shaban M. Screening of Mild Cognitive Impairment in Patients with Parkinson's Disease Using a Variational Mode Decomposition Based Deep-Learning. 2023 11th International IEEE/EMBS Conference on Neural Engineering (NER). 2023;doi:10.1109/NER52421.2023.10123759.

[49] Park CJ, Eom J, Park KS, Park YW, Chung SJ, Kim YJ, et al. An Interpretable Multiparametric Radiomics Model of Basal Ganglia to Predict Dementia Conversion in Parkinson's Disease. npj Parkinson's Disease. 2023;9(1):1–10. doi:10.1038/s41531-023-00566-1.

[50] Peng B, Zhou Z, Geng C, Tong B, Zhou Z, Zhang T, et al. Computer Aided Analysis of Cognitive Disorder in Patients with Parkinsonism Using Machine Learning Method with Multilevel ROI-based Features. In: Proceedings - 2016 9th International Congress on Image and Signal Processing, BioMedical Engineering and Informatics, CISP-BMEI 2016. Institute of Electrical and Electronics Engineers Inc.; 2017. p. 1792–1796.

[51] Picardi C, Cosgrove J, Smith SL, Jamieson S, Alty JE. Objective Assessment of Cognitive Impairment in Parkinson's Disease Using Evolutionary Algorithm. In: European Conference on the Applications of Evolutionary Computation. vol. 10199 LNCS. Springer Verlag; 2017. p. 109–124.

[52] Rallabandi VPS, Seetharaman K. Machine Learning-Based Classification of Dementia Types: MRI Study. In: Proceedings - International Conference on Artificial Intelligence and Smart Systems, ICAIS 2021. Institute of Electrical and Electronics Engineers Inc.; 2021. p. 109–114.

[53] Ramezani M, Mouches P, Yoon E, Rajashekar D, Ruskey JA, Leveille E, et al. Investigating the Relationship between the SNCA Gene and Cognitive Abilities in Idiopathic Parkinson's Disease Using Machine Learning. Scientific Reports. 2021;11(1):1–10. doi:10.1038/s41598-021-84316-4.

[54] Ricciardi C, Amboni M, Santis CD, Ricciardelli G, Improta G, D'Addio G, et al. Machine Learning Can Detect the Presence of Mild Cognitive Impairment in Patients Affected by Parkinson's Disease. 2020 IEEE International Symposium on Medical Measurements and Applications (MeMeA). 2020;doi:10.1109/MEMEA49120.2020.9137301.

[55] Russo M, Amboni M, Barone P, Pellecchia MT, Romano M, Ricciardi C, et al. Identification of a Gait Pattern for Detecting Mild Cognitive Impairment in Parkinson's Disease. Sensors. 2023;23(4):1985. doi:10.3390/S23041985/S1.

[56] Salmanpour MR, Shamsaei M, Saberi A, Setayeshi S, Klyuzhin IS, Sossi V, et al. Optimized Machine Learning Methods for Prediction of Cognitive Outcome in Parkinson's Disease. Computers in Biology and Medicine. 2019;111:103347. doi:10.1016/J.COMPBIOMED.2019.103347.

[57] Schrag A, Siddiqui UF, Anastasiou Z, Weintraub D, Schott JM. Clinical Variables and Biomarkers in Prediction of Cognitive Impairment in Patients with Newly Diagnosed Parkinson's Disease: A Cohort Study. The Lancet Neurology. 2017;16(1):75. doi:10.1016/S1474-4422(16)30328-3.

[58] Shibata H, Uchida Y, Inui S, Kan H, Sakurai K, Oishi N, et al. Machine Learning Trained with Quantitative Susceptibility Mapping to Detect Mild Cognitive Impairment in Parkinson's Disease. Parkinsonism and Related Disorders. 2022;94:104–110. doi:10.1016/j.parkreldis.2021.12.004.

[59] Shin NY, Bang M, Yoo SW, Kim JS, Yun E, Yoon U, et al. Cortical Thickness from MRI to Predict Conversion from Mild Cognitive Impairment to Dementia in Parkinson Disease: A Machine Learning-Based Model. Radiology. 2021;300(2):390. doi:10.1148/RADIOL.2021203383.

[60] Suo X, Lei D, Li N, Li J, Peng J, Li W, et al. Topologically Convergent and Divergent Morphological Gray Matter Networks in Early-Stage Parkinson's Disease with and without Mild Cognitive Impairment. Human Brain Mapping. 2021;42(15):5101–5112. doi:10.1002/HBM.25606.

[61] Suwalska A, Siuda J, Kocot S, Zmuda W, Rudzinska-Bar M, Polanska J. Activation Maps of Convolutional Neural Networks as a Tool for Brain Degeneration Tracking in Early Diagnosis of Dementia in Parkinson's Disease Based on Magnetic Resonance Imaging. Signal, Image and Video Processing. 2023; p. 1–7. doi:10.1007/S11760-023-02643-7.

[62] Talkar T, Manxhari C, Williamson JR, Smith KM, Quatieri TF. Speech Acoustics in Mild Cognitive Impairment and Parkinson's Disease With and Without Concurrent Drawing Tasks. In: Proceedings of the Annual Conference of the International Speech Communication Association, INTERSPEECH. vol. 2022-September. International Speech Communication Association; 2022. p. 2258–2262.

[63] Tao S, Wang Y, Cai H, Lv Z, Kong L, Lv W. Recognition of Parkinson's Disease and Parkinson's Dementia Based on Gait Analysis and Machine Learning. In: Third International Symposium on Computer Engineering and Intelligent Communications (ISCEIC 2022). vol. 12462. SPIE; 2023. p. 593–601.

[64] Tsiouris KM, Konitsiotis S, Koutsouris DD, Fotiadis DI. A Risk Stratification Model for Early Cognitive Impairment after Diagnosis of Parkinson's Disease. In: XV Mediterranean Conference on Medical and Biological Engineering and Computing – MEDICON 2019. vol. 76. Springer; 2020. p. 653–660.

[65] Tülay EE, Yıldırım E, Aktürk T, Güntekin B. Classification of Parkinson's Disease with Dementia Using Phase Locking Factor of Event-Related Oscillations to Visual and Auditory Stimuli. Journal of Neural Engineering. 2023;20(2):026025. doi:10.1088/1741-2552/ACC612.

[66] Xu H, Liu Y, Wang L, Zeng X, Xu Y, Wang Z. Role of Hippocampal Subfields in Neurodegenerative Disease Progression Analyzed with a Multi-Scale Attention-Based Network. NeuroImage: Clinical. 2023;38:103370. doi:10.1016/J.NICL.2023.103370.

[67] Yu Z, Pang H, Yu H, Wu Z, Ding Z, Fan G. Segmental Disturbance of White Matter Microstructure in Predicting Mild Cognitive Impairment in Idiopathic Parkinson's Disease: An Individualized Study Based on Automated Fiber Quantification Tractography. Parkinsonism & Related Disorders. 2023;115. doi:10.1016/j.parkreldis.2023.105802.

[68] Zhang C, Wu C, Zhang H, Dou W, Li W, Sami MU, et al. Disrupted Resting-state Functional Connectivity of the Nucleus Basalis of Meynert in Parkinson's Disease with Mild Cognitive Impairment. Neuroscience. 2020;442:228–236. doi:10.1016/j.neuroscience.2020.07.008.

[69] Zhang J, Li Y, Gao Y, Hu J, Huang B, Rong S, et al. An SBM-based Machine Learning Model for Identifying Mild Cognitive Impairment in Patients with Parkinson's Disease. Journal of the Neurological Sciences. 2020;418. doi:10.1016/j.jns.2020.117077.

[70] Zhang J, Gao Y, He X, Feng S, Hu J, Zhang Q, et al. Identifying Parkinson's Disease with Mild Cognitive Impairment by Using Combined MR Imaging and Electroencephalogram. European Radiology. 2021;31(10):7386–7394. doi:10.1007/S00330-020-07575-1.
