# Supplementary material for: Machine learning for the detection and diagnosis of cognitive impairment in Parkinson’s Disease: A systematic review
Source: PLoS One. 2024 May 16;19(5):e0303644. doi: 10.1371/journal.pone.0303644 (PMC11098383; doi:10.1371/journal.pone.0303644)
Supplement: S4 File — Performance and characteristics of ML methods used for each data modality in reviewed literature. (PDF) [file pone.0303644.s004.pdf]

Machine Learning for the Detection and Diagnosis of Cognitive Impairment in Parkinson's Disease: A Systematic Review - Supplementary Material

Callum Altham*[1], Huaizhong Zhang[1], and Ella Pereira[1]

[1]Department of Computer Science, Edge Hill University, St. Helens Road, Ormskirk, L39 4QP, Lancashire, United Kingdom

Table S4: Performance and characteristics of ML methods used for each data modality in reviewed literature.
ML Method(s) = ML methods used to achieve the highest per-study performance. Performance = Averaged performance(s) from ML models, studies with singularly used metrics include all used metrics

Modality	Data Sources	Activities	ML Method(s)	Performance
Imaging	Collected, PPMI	Diagnosis, Prediction, Biomarker Identification, Differential Diagnosis	SVM, Tree, Regression, ANN, K-NN, NB	Accuracy = 81.60%
Clinical Characteristics	PPMI, Collected, Cohort, NBBK	Diagnosis, Prediction, Biomarker Identification	Tree, Regression, SVM, ANN, Hybrid	Accuracy = 82.92%
EEG	Collected	Diagnosis, Biomarker Prediction, Differential Diagnosis	Tree, K-NN, ANN, SVM, DA, DR	Accuracy = 88.01%
Neuropsychological Profile	Collected, PPMI, NBBK, Cohort	Diagnosis, Prediction, Differential Diagnosis	Tree, SVM, DA, ANN, K-NN	Accuracy = 89.67%
Gait & Movement	Collected	Diagnosis, Prediction, Biomarker Identification	Tree, SVM, GMM, CGP	Accuracy = 86.33%
Genetic & Epigenetic	PPMI, Collected, Cohort	Diagnosis, Prediction, Biomarker Identification	Tree, Regression	Accuracy = 81.79%
Other	PPMI, Cohort, Collected	Diagnosis, Prediction	Tree, Ensemble	Accuracy = 76.0%
Demographics	Collected, PPMI	Diagnosis, Prediction, Differential Diagnosis	Tree, SVR, ANN, K-NN	Accuracy = 87.83%
Sleep Behaviour	NBBK, PPMI	Diagnosis, Differential Diagnosis	Tree, Hybrid, Regression	Accuracy = 79.23%
Medical History	PPMI, Cohort	Diagnosis, Prediction	Tree, Regression	Accuracy = 73.6%
Motor Symptoms	NBBK, PPMI	Diagnosis, Prediction	Tree, Hybrid	Accuracy = 84.94%

Continued on the next page

*Corresponding Author: althamc@edgehill.ac.uk

Modality	Data Sources	Activities	ML Method(s)	Performance
Blood Biomarkers	Collected, Cohort	Diagnosis, Prediction, Biomarker Identification	SVM, ANN, Regression, Tree	Accuracy = 88.2%
Speech Features	Collected	Diagnosis, Biomarker Identification	SVM, GMM	Accuracy = 65.3%
Non-Motor Symptoms	NBBK, PPMI	Diagnosis	Tree, Hybrid	Accuracy = 84.94%
CSF	PPMI	Diagnosis, Prediction	SVM, SVR, LoR	PCC = 0.44, (MAE, RMSE) = (0.076, 0.542), AUC = 0.80

S4 Legend:

- **ANN:** Artificial Neural Network
- **AUC:** Area under the ROC Curve
- **CGP:** Cartesian Genetic Programming
- **CSF:** Cerebrospinal Fluid
- **DA:** Discriminant Analysis
- **DR:** Dimensionality Reduction
- **EEG:** Electroencephalogram
- **GMM:** Gaussian Mixture Model
- **K-NN:** K Nearest Neighbour
- **LoR:** Logistic Regression
- **MAE:** Mean Absolute Error
- **ML:** Machine Learning
- **NB:** Naïve Bayes
- **NBBK:** National BioBank of Korea
- **PCC:** Pearson's Correlation Coefficient
- **PPMI:** Parkinson's Progression Markers Initiative
- **RMSE:** Root Mean Square Error
- **SVM:** Support Vector Machine
- **SVR:** Support Vector Regression
