# Supplementary material for: Machine learning for the detection and diagnosis of cognitive impairment in Parkinson’s Disease: A systematic review
Source: PLoS One. 2024 May 16;19(5):e0303644. doi: 10.1371/journal.pone.0303644 (PMC11098383; doi:10.1371/journal.pone.0303644)
Supplement: Attachment — Submitted filename: response-to-review.pdf [file pone.0303644.s006.pdf]

Dear Editors and Reviewers,

We would like to thank the reviewers for their detailed and constructive feedback. Our responses to the reviewers' comments are provided below. The corresponding modified text is highlighted in the track changes version of the manuscript.

Reviewers' Comments	Our Responses
Reviewer 1: *In this article, authors presented a systematic and narrative review of the machine learning techniques, data sources and modalities used for detection and diagnosis of cognitive impairment in Parkinson's Disease. 70 studies were included in this review, with the most relevant information extracted from each. From each study, strategy, modalities, sources, methods and outcomes were extracted. This paper clearly provides important information for the versatility of machine learning in analysing a range of modalities for the detection and diagnosis of cognitive impairment in Parkinson's Disease, including imaging, EEG, speech and more, yielding notable diagnostic accuracy.*	
1.1 Considering the nature of systematic and narrative review, please cite the following papers, presenting the association between quantitative susceptibility mapping and cognition in Parkinson's disease in the Introduction:<br>*10.1002/mds.27717*<br>*10.1002/mds.28077*<br>*10.3389/fnins.2022.938092*	**Response to 1.1** – These additional papers have now been cited within the introduction on line 33, as well as citations 31-34 in the references list.
1.2 In Figure 2, the characters are too small to recognize the words.	**Response to 1.2** – Figure 2 has now been split into two constituent figures and now exist as figures 2 and 4. All other document figures have now been converted into an EPS format as well as changes to the overall figure structure and textual elements (including size) to enhance readability visualization and understanding of numerical values before being processed through the recommended PACE system to ensure suitability. These figures have all also been changed to ensure that all figures follow a similar style of graphical representation and format.

Reviewer 2: *Dear Authors,*
your study focuses on a systematic literature review that aims to test the potential of artificial intelligence algorithms in a highly prominent clinical area, the prediction and diagnosis of non-motor symptomatology in Parkinson's Disease. Although I consider the topic to be of high interest and in accordance with the direction of today's literature, some points need to be considered to improve your work.

2.1 In the Abstract Background section the aim of the study should be clarified.

Response to 2.1 – The abstract text in the manuscript has now been updated to provide a description of the aim consistent with the descriptions given later on in the article text with the addition of “This work aims to provide an overview of published studies applying machine learning to detecting and diagnosing cognitive impairment, evaluate the feasibility of implemented methods, their impacts, and provide suitable recommendations for methods, modalities and outcomes.”.

2.2 In the Abstract Results section, please change "a range of modalities..." in " a wide range of different data modalities..".

Response to 2.2 – The manuscript has been updated to include this change of wording with the abstract now reading “Our review demonstrates the versatility of machine learning in analysing a wide range of different modalities for the detection and diagnosis…” .

2.3 In the Introduction section in line 2 please change "the most common manifestation of Parkinsonism" in "the most common neurodegenerative disorder", and in line 3 change "unintended, uncontrollable movements" in "dyskinesia".

Response to 2.3 – The manuscript has been updated to include these changes to terms and descriptions on lines 2 and 3.

2.4 This point refers to the paragraph on Parkinson's disease in the Background section. Although the clinical content reported is correct, the message from the text is that this disease is primarily cognitive, as if it was a dementia, as opposed to Parkinson's being a motor disease.
Therefore, I recommend that you rewrite this section emphasizing the concept that Parkinson's is a neurodegenerative motor disease. It is important to emphasize that the manifestations at onset are motor in nature, stressing which are the main motor symptoms (rigidity, tremor, and bradykinesia), leaving as the last description the non-motor symptomatology as a manifestation both cognitive, related to the domain of execution functions, attention, visuospatial abilities, and language, given the fact that dopamine is also related to the cognitive circuit, but also anosmia, sialorrhea, and swallowing.

Response to 2.4 – Based on reviewer advice, the original Parkinson's Disease section of the background has now been updated to frame Parkinson's as a disease primarily motor in nature rather than solely cognitive based, with discussions of cognitive based non-motor symptomology that are also present as the disease progresses now mentioned after discussion of the characteristic motor elements of the disease. These additions to the manuscript can be found on lines 64-82.

2.5 In the Background Cognitive Impairment section, after lines 96-98 in which you describe neuropsychological screening tools, I recommend that you add a sentence that tells the higher diagnostic power of MoCA than MMSE, because the first must be used in clinical practice for cognitive screening in PD.

Response to 2.5 – The manuscript text has been updated to include this additional context regarding the MoCA and MMSE, with the sentence "The MoCA is generally considered to perform a have higher diagnostic power compared to the MMSE due to its broader cognitive domains used in assessment, heightened sensitivity to MCI and early dementia, and better adjustment for education levels, making it a superior tool in detecting subtle cognitive changes." Added to lines 105-110.

2.6 In the Background Machine Learning section, after "ML has emerged as a valuable tool in a variety of healthcare applications, including the detection of PD" you cite a paper that uses data retrieved from clinical questionnaires and instrumental data, which are not very ecological. So I think you can add this reference to the previous one:

Palmirotta, Cinzia, et al. "Unveiling the Diagnostic Potential of Linguistic Markers in Identifying Individuals with Parkinson's Disease through

Response to 2.6 – This additional review paper has been cited at the end of the relevant sentence on line 118, and now exists as reference 66 in the overall reference list.

Artificial Intelligence: A Systematic Review." Brain Sciences 14.2 (2024): 137.

which demostrates how ecological language tasks could predict PD. This states how either ecological or instrumental data combined with ML can predict PD.

2.7 In the Background Supervised Learning section, in line 116 before ending the period, I suggest adding "to solve either regression or classification tasks." Since in this section you rightly describe supervised models associating them with regression tasks, classification tasks, or both; after the end of the sentence first mentioned and corrected, it seems more correct to me to explain both tasks, their differences, and how a regression task can become a classification one.
The regression task was explained in lines 133-136, so it could be moved by reframing the concept referring not to methods but to tasks, while the classification task should be added entirely because it was never explained.

Response to 2.7 – This additional text suggestion has now been added to the manuscript text on line 129. Additionally, the background supervised learning section has now been re-written to place a focus on the performing of both classification and regression tasks by these models, with the regression task description now moved to earlier in the section and an additional description of classification tasks provided alongside it before the text continues to discuss types of supervised learning methods. This moved and additional text can be found on lines 132 – 144.

2.8 In line 149, please add before the period "respectively for, classification and regression tasks."

Response to 2.8 – This additional text has been added to the manuscript on line 172-173 and now ends the sentence with *"respectively for, classification and regression tasks."*

2.9 In background Unsupervised Learning, in lines 187-189, you explain PCA but I think that it should be better explained, because important information are missing.

Response to 2.9 – The manuscript text has now been updated to contain a more detailed description of PCA containing additional information regarding the mechanics of PCA alongside the usage of PCA beginning with “PCA is a technique…” on lines 212-216.

2.10 In the Background: Deep Learning section in lines 207-209 state that deep learning is so called because the models consist of multiple layers. Although deep learning models are more complex in terms of architecture, as you defined, the term deep is associated with the ability of such models to extract trainable, hierarchically organized features directly from raw data, thus not requiring the feature extractor block, i.e., engineered features extracted upstream and not trainable, before the definition of the model itself. For this reason, the architecture is more complex, i.e. formed by multiple layers. Please, rewrite this definition.

Response to 2.10 – Based on reviewer advice, the deep learning section of the background has now been updated to change the definition of "deep neural networks" to now align with this definition regarding the lack of a feature extraction block and the ability of such networks to extract features from raw data, creating a more complex network architecture. This redefined definition can be found beginning with "DL differs significantly from traditional…" on lines 234 – 244.

2.11 In lines 216-218, when you explain the limitations of deep learning models, I think that the need for very huge datasets for training is worth mentioning.

Response to 2.11 – The deep learning background section has now been updated to contain this reference to the issue posed by the need for large datasets by mentioning the common dataset ImageNet containing 3.2 million samples ("…comes with challenges such as the need for datasets containing large numbers of labelled samples, with common deep learning datasets such as the ImageNet dataset including over 3.2 million samples…") on lines 251-253.

2.12 In the Background Ensemble learning, in lines 220-225 you explain ensemble learning benefits, the overfitting reduction is worth mentioning.

Response to 2.12 – The background ensemble learning section has now been updated to include discussions of the benefit of ensemble learning for overfitting reduction on lines 265-268.

2.13 In Table 1, the LR symbol is associated to Logistic Regression, as I understand from the description, but in the Background supervised learning section it is associated to Linear Regression. This is ambiguous, please clarify this symbol association.

Response to 2.13 – This was an oversight on our part that was left un-noticed. These two symbol associations have now been updated with LoR indicating Logistic Regression and LiR indicating Linear Regression. A sentence has also been added to the background machine learning section on lines 161-164 to discuss logistic regression alongside adding another table row to Table 1 on page 7 to discuss Linear Regression. Each occurrence of the symbol LR in the supplementary table and the manuscript have now been updated to contain the relevant symbol of LoR or LiR depending on the usage.

2.14 In the Search strategy section, Table S1 is mentioned. In Pubmed, strategy reviews are not included, what for IEEE xplore, Scopus and Science Direct? Reviews removal is not mentioned either in Figure 1 or in the text.

Response to 2.14 – IEEE Xplore [1] and Science Direct [2] do not directly allow for the removal of references during the search query (see references) and instead these papers have to be filtered after the point of search. These review papers were originally included in the overall number of papers removed during the screening stage of Figure 1 but consultation with search records means that Figure 1 has now been updated to contain a direct statement regarding the removal of these specific papers. With regard to Scopus, the ability to remove search queries was overlooked in the original query, but this feature does exist in Scopus' search system. Therefore, the original search query was retrospectively re-ran with this new additional restriction to determine the number of review papers that formed the original search query and were removed in the new version of the query. Each of the numbers that formed the PRISMA statement in Figure 1 were then updated to include these changes to the number of retrieved publications as well as the stages in which papers were removed. The 'Literature Review Eligibility' section of the manuscript on page 11 was also updated to include these new figures as well as the removal of the review papers. Additionally, the supplementary material Table S1 was updated to include the new version of the Scopus search query that includes this new document type restriction.

2.15 In the Data Extraction section, you describe the information obtained from papers, you mentioned Data Source but this information is not present in Table S3. Is it a typos?
Furthermore, I think that you should also extract Validation-and-testing method, ie. crossvalitation? Nested-crossavalisation? Train test split? and add it an column in Table S3.

Response to 2.15 – Data source was incorrectly removed from the information in S3 during efforts to ensure the table was correctly formatted to fit within the page dimensions. The supplementary material has now been updated to contain this column in table S3 and the page layout made landscape to allow this column to fit. Additionally, a new column has been added to table S3 named "validation" containing description of the validation and testing methods used by each individual literature with an additional item added to the data extraction section to reflect this.

2.16 I recommend to place Literature Review Eligibility section before Data Sources section, because it belongs to Observations and Findings.

Response to 2.16 – This section has now been relocated to the beginning of the observation and findings section before the data sources section and can now be found on lines 406-415.

2.17 Figure 2 is not described or mentioned in the text, only from Line 390 but is in brackets.

Response to 2.17 – The manuscript text has now been updated to include a description of the contents of figure 2 within the text and is found in the data modalities section on lines 440-441.

2.18 Lines 405-407, should be placed at the start of the results section.

Response to 2.18 – These lines of text have now been moved from the "Machine Learning Techniques" section to the "*ML Techniques Applied in CI Detection and Diagnosis" section with the two sentences forming lines 405-407 now present on lines 496-497 and 505-507.*

2.19 Figure 3 is not described in the text.

Response to 2.19 – The manuscript text has now been updated to include a description of figure 3 within the text and is found in the "Machine Learning Techniques" section on lines 463-465.

2.20 Please don't cite figures in brackets

Response to 2.20 – All figure citations or descriptions have now been changed to no longer include the use of brackets.

2.21 Figure's quality should be improved.

Response to 2.21 – Figure 2 has now been split into two constituent figures and now exist as figures 2 and 4. All other document figures have now been converted into an EPS format as well as changes to the overall figure structure and textual elements (including size) to enhance readability visualization and understanding of numerical values before being processed through the recommended PACE system to ensure suitability. These figures have all also been changed to ensure that all figures follow a similar style of graphical representation and format.

2.22 A legend after Table S3 should be added

Response to 2.22 – Table S3 has now been updated to contain a more detailed caption regarding the contents of the table and the elements within each column. A legend has now also been placed after the end of Tables S3 and S4 containing details of the meaning of all acronyms present within the tables in the supplementary material.

2.23 In Data sources, Study Activities, Data Modalities, Machine laerning techiniques, Performance Metrics sections after indicating the number of papers (N=value) next to each result, the references of all papers associated with that result should be included.

Response to 2.23 – All occurrences of a number of papers next to a result in each of these sections have now been updated to include the references for the papers contributing to this result after. Some statements are not provided with references if they are then further split into individual smaller results, with these smaller results having themore relevant references instead.

2.24 I suggest adding another supplementary Table in which you describe Data Modalities, i.e. by putting along rows the different modalities and in the next columns all kinds of data belonging to that category. The reference in the text could be placed in Data Extraction, indicating a description of your data modality

Response to 2.24 – A table containing information per data modality was originally available in the manuscript as table 2 however the caption was deemed to not be descriptive of this and therefore it may have been overlooked. However, this table has since been updated to contain additional information to ensure that it provides the correct amount of detail regarding individual data modalities. The inclusion of this extra information meant that the table no longer efficiently fit into the manuscript text and has now been moved to the supplementary material as table S4, with the supporting information section updated to reflect this and a new statement added to the data extraction section on lines 365-366.

2.25 In the Results Supervised Learning section, add references to papers contributing to that result after (N=value).

Response to 2.25 – All occurrences of a number of papers next to a result in the results section have now been updated to contain the references for all papers contributing to this result after. Some statements are not provided if they are further split into individual smaller results, with these smaller results having the more relevant references instead.

2.26 In the Results Classification section please, before every reference, add Authors et al. not only the citation.

Response to 2.26 – All sections of the manuscript have been rechecked and all referenced authors within the results section have now been updated to contain the "authors et al." description for each individual paper at the point of discussion.

2.27 AUC and accuracy metrics are sometimes presented as percentages and other times as float numbers; please always use the same wording in the textual results section as in Table S3

Response to 2.27 – AUC and accuracy metrics were originally written based on the format was provided within the actual research paper referenced causing this mismatch between different presentations of metrics. We understand that this may cause some confusion and therefore all mentions of accuracy within the manuscript and supplementary are now presented in a percentage format whilst all AUC metrics are presented as a float number.

2.28 In line 826, what is "Per Fig.3"? Is it a typo?

Response to 2.28 – This text was originally written to indicate the reader to look at figure 3 however it is understood this may be difficult to understand to other English speakers. This text has now been rewritten to state "As shown in fig. 3" on line 522 to better indicate that this text is talking in regard to this specific figure.

2.29 In the Discussion section before starting, I suggest resuming the purpose of the work and a brief recap of the most important findings so that we can then tie the discussion together.

Response to 2.29 – At the beginning of the discussion section, additional text has now been added to reaffirm the purpose of the work as well as discussing the most important findings of the work before leading into the discussion section. This new text can be found at the start of the discussion section on lines 867-882.

2.30 In line 902, you can also add the benefit of fine tuning in brief.

Response to 2.30 – The manuscript text has now been updated in the "Condition Classification and Prediction" section to contain this additional benefit of transfer learning and is found on lines 991-997.

2.31 In supporting information Table S2 and Table S3 are not mentioned.	**Response to 2.31** – These tables have now been included within the supplementary material statement at the end of the manuscript on pages 25-26, with the supplementary material now updated to contain tables S1-S4 alongside the PRISMA checklist as item S5.
2.32 Please check for any typos and English grammar.	**Response to 2.32** – The manuscript has been thoroughly proofread and checked for any typos or English grammar issues that may have been overlooked, resulting in some small textual changes to the manuscript.

References

[1] ***https://ieeexplore.ieee.org/Xplorehelp/searching-ieee-xplore/command-search#summary-of-data-fields***

[2] ***https://service.elsevier.com/app/answers/detail/a_id/25974/supporthub/sciencedirect/***
